# A new phototherapy regimen during winter as an add-on therapy, coupled with oral vitamin D supplementation, for the long-term control of atopic dermatitis: study protocol for a multicentre, randomized, crossover, pragmatic trial – the PRADA trial

**DOI:** 10.1186/s13063-019-3276-9

**Published:** 2019-03-25

**Authors:** Catherine Droitcourt, Sébastien Barbarot, Annabel Maruani, Laure Darrieux, Laurent Misery, Emilie Brenaut, Henri Adamski, Cécile Chabbert, Annie Vermersch, Marie Weiborn, Julien Seneschal, Alain Taïeb, Patrice Plantin, Hervé Maillard, Alice Phan, François Skowron, Manuelle Viguier, Delphine Staumont-Salle, Audrey Nosbaum, Angèle Soria, Annick Barbaud, Emmanuel Oger, Alain Dupuy, Groupe de Recherche sur l’Eczéma Atopique de la Société Française de Dermatologie (GREAT)

**Affiliations:** 1 Univ Rennes, Rennes, France; 2 Department of Dermatology, University Hospital Center of Rennes, Pontchaillou Hospital, 2 rue Henri le Guilloux, 35000 Rennes, France; 3 INSERM, CIC 1414, 35000 Rennes, France; 4 EA 7449 REPERES “Pharmaco-epidemiology and Health Services Research”, Univ Rennes, 35000 Rennes, France; 5 Department of Dermatology, University Hospital Center of Nantes, 44000 Nantes, France; 6 Department of Dermatology, University Hospital Center of Tours, 37000 Tours, France; 7 Department of Dermatology, Hospital Center of Saint-Brieuc, 22000 Saint-Brieuc, France; 8 Department of Dermatology, University Hospital Center of Brest, 29000 Brest, France; 9 Department of Dermatology, Hospital Center of Perigueux, 24000 Perigueux, France; 10 Department of Dermatology, Hospital Center of Valenciennes, 62000 Valenciennes, France; 11 Department of Dermatology, University Hospital Center of Bordeaux, 33000 Bordeaux, France; 12 Department of Dermatology, Hospital Center of Quimper, 29000 Quimper, France; 13 Department of Dermatology, Hospital Center of Le Mans, 72000 Angers, France; 14 Department of Dermatology, University Hospital Center of Lyon, 69000 Lyon-Bron, France; 15 Department of Dermatology, Hospital Center of Valence, 26000 Valence, France; 16 Department of Dermatology, University Hospital Center of Reims, 51000 Reims, France; 17 Department of Dermatology, University Hospital Center of Lille, 59000 Lille, France; 18 Department of Dermatology, University Hospital Center of Lyon, 69000 Lyon Sud, France; 19 Department of Dermatology and Allergology, University Hospital Center of Paris-Tenon, 75020 Paris, France

**Keywords:** Atopic dermatitis, vitamin D, phototherapy, long-term control, add-on therapy, pragmatic trial

## Abstract

**Background:**

Atopic dermatitis is a highly prevalent, chronic, relapsing disease in both adults and children. On the severity spectrum, lower-end patients benefit from small amounts of topical anti-inflammatory treatments (TAT), whereas higher-end patients need systemic immunosuppressants; in-between patients are treated with TAT and phototherapy. The major therapeutic challenge in this population is the long-term control of disease activity, and the current TAT-based pro-active strategy does not meet all their needs. Immunosuppressants are used as long-term control add-on treatments, but they are restricted to the most severely affected patients because of safety concerns. In addition, neither immunosuppressants nor other strategies have been properly evaluated in the long term despite long-term control having been acknowledged as one of the most important core outcome domains to be targeted in atopic dermatitis trials. Safe add-on therapies, rigorously evaluated for long-term control of the disease, are therefore needed. Phototherapy and vitamin D supplementation are both good candidates.

**Methods:**

This is a multicenter, national, randomized, superiority, crossover trial testing add-on phototherapy (one winter under spaced sessions of phototherapy and one winter under observation) among subjects receiving standard care (i.e., TAT). On the same population, we will test the long-term control provided by oral supplementation of vitamin D versus placebo in a randomized, superiority, double-blind, parallel-group trial. The primary outcomes are (1) repeat measures of the PO-SCORAD severity score over 1 year and (2) cumulate consumption of TAT (number of tubes) during the winter. They will be tested following a hierarchical testing procedure. The secondary outcomes will be measures repeated over 2 years of investigator-based severity scores, patient-reported severity and quality of life scores, serum vitamin D levels, weeks during which the disease is well-controlled, inter-visit cumulate consumption of TAT, and synthetic patient-reported satisfaction at the end of each winter.

**Discussion:**

This study includes two separate 2-year pragmatic trials designed to evaluate the efficacy of vitamin D supplementation and pro-active phototherapy for primary care atopic dermatitis patients receiving TAT on long-term control of disease activity. The experimental design enables the study of both interventions and exploration of the interaction between vitamin D and phototherapy. A pragmatic trial is particularly suited to the assessment of long-term control. This study explores the possibility of new and safe therapeutic strategies for the control of long-term atopic dermatitis, and is an example of efficacy research that is unlikely to be sponsored by industrialists. A potentially effective low-cost therapeutic strategy for long-term control is essential for patients and public health.

**Trial registration:**

ClinicalTrials.gov Identifier: NCT02537509, first received: 1 September 2015.

**Electronic supplementary material:**

The online version of this article (10.1186/s13063-019-3276-9) contains supplementary material, which is available to authorized users.

## Background

Atopic dermatitis (AD) is a common pruritic inflammatory skin disease that classically has a chronic course of flares and remissions. AD is a highly prevalent skin disorder, in both adults (2–10%) [1–4] and children (15–20%) [1, 5–7], and there is some evidence of an increase in AD prevalence in some regions of the world, including Western Europe [8]. Children with persistent and/or severe disease have increased AD persistence in adulthood [9].

On the severity spectrum, lower-end patients benefit from emollients and small amounts of topical corticosteroids, whereas higher-end patients need systemic immunosuppressants; in-between patients are treated with topical corticosteroids and tacrolimus (topical anti-inflammatory treatments; TAT) and phototherapy. The management of AD requires efficient control of the flares. TAT are important anti-inflammatory drugs used in AD flares. Topical corticosteroids applied on inflammatory lesional skin are widely used as a first-line treatment for disease flares. They have been evaluated in a wide variety of preparations in more than 110 different randomized controlled trials [10]. Topical calcineurin inhibitors are a second class of TAT drugs that have also proved efficacious against AD flares [11], and are administered alone or in combination with topical steroids. Phototherapy is widely used as a second-line treatment for the short-term control of moderate-to-severe AD with demonstrated efficacy, with intensive schedules of 2–7 sessions a week for 2 or 3 months [12, 13].

These chronic in-between AD patients need large amounts of TAT to maintain control of disease activity. They certainly have the most markedly unmet medical need, with a high impact on quality of life compared to other chronic inflammatory skin diseases [14]. The major therapeutic challenge in this population is the long-term control (LTC) of disease activity, and the current TAT-basedpro-active strategy (intermittent applications of TAT after flares on previously affected skin, over long-term periods) does not meet all the needs. There is also consistent evidence of subclinical inflammation in skin of normal appearance and in the treated skin of AD patients. Similarly, there is evidence that treatments might improve this sub-clinical inflammation, prevent new flares or decrease the risk of new flares in long-termfollow-up [15]. Current treatments for LTC include strategies such as daily emollient application, intermittent use of TAT (TAT-based strategy: once or twice a week [15–18]), avoidance strategies, dietary interventions, and immunosuppressive drugs.

‘Add-on’ therapies, which are superimposed on standard TAT, seem promising for LTC. Systemic immunosuppressants are used as LTC add-on treatments, but they are reserved for the most severely affected patients because of safety concerns. In addition, neither immunosuppressants nor other strategies (with the exception of desensitization) [19] have been evaluated in the long term. LTC is one of the most important core outcome domains to be targeted in AD trials according to the HOME (Harmonising Outcome Measures for Eczema) recommendations [20]. Safe add-on therapies, rigorously evaluated for disease LTC, are therefore needed.

Phototherapy is a good candidate for the ‘in-between’ AD population, being widely used as a second-line treatment in AD with demonstrated efficacy [12]. However, only short-term control has been evaluated, and only intensive schedules of two or three sessions per week have been tested and are used in current practice. A novel phototherapy regimen enabling a trade-off between disease control, ultraviolet (UV)-induced risks, and patient acceptability is required for LTC of the disease. No serious short-term adverse events were collected in the review by Garritsen et al. [12], where the most common adverse event was xerosis.

Vitamin D supplementation is another good candidate for the ‘in-between’ AD population, with several studies having shown lower serum levels of vitamin D to be correlated with more severe AD [21–26]. However, in these types of association studies, confounding factors such as solar exposure and socio-economic level, can be a major limitation. These observational studies are supported by some evidence that vitamin D could play a role in the different stages of AD pathogenesis [27, 28]. A meta-analysis on trials testing vitamin D supplementation yielded conflicting results, leaving its therapeutic efficacy undecided for short-term control and unknown for LTC [25].

We hypothesize that spaced-out phototherapy sessions during winter (exploratory trial) and vitamin D as an add-on therapy (main trial) could be beneficial to improve LTC of disease activity among AD patients.

## Methods/Design

### Objectives

Since both phototherapy and vitamin D supplementation are good candidates for LTC of disease activity in AD, we propose to study these two treatments jointly for the LTC of AD.

### Trial design

The study is a pragmatic, multicenter, national, randomized (1:1), superiority, crossover (two periods, two sequences) trial lasting 2 years (including two successive winters), and exploring add-on phototherapy versus observation in subjects receiving standard care (i.e., TAT). In the same population, we will be testing the LTC provided by oral supplementation of vitamin D versus placebo in a randomized, superiority, double-blind, parallel-group trial. Pragmatic trials [29] are the best-suited designs for studying add-on therapies in the context of long-term AD disease. The main pragmatic options are the following: recruitment in a primary care population, standard vitamin D supplementation, regimen not adapted to serum levels, strictly ‘as usual’ TAT use, and no recommendation on changes in lifestyle.

### Study setting

The study will be conducted in 16 French hospital centers with a network of primary care dermatologists in France.

### Participants and eligibility criteria

#### Inclusion criteria

Eligibility criteria: patients with AD (Hanifin and Rajka criteria), aged 15 or over, with > 2 years of disease evolution, moderate-to-severe disease (Investigator Global Assessment > 2), seasonality in disease severity (improvement in summer), and fewer than 100 previous phototherapy sessions in their lifetime. Eligible patients are to have received TAT for at least 12 weeks and to have symptoms requiring an intensification of the therapy, and they are to have easy access to a phototherapy cabin (widely available in primary care dermatology private practices in France).

#### Exclusion criteria

Exclusion criteria: patients with AD known to be aggravated by UV exposure; indication for a systemic immunosuppressant in the next 2 years; any cause of contra-indication for vitamin D supplementation: granulomatosis flare, primary hyperparathyroidism; clinical suspicion of hypercalciuria; any contra-indication for artificial or solar photo-exposure including genetic disease predisposing to skin cancer, any history of skin cancer (melanoma, squamous and basal skin cancers), lupus, dermatomyositis, any other photosensitizing skin disease, photosensitizing medication; and more than 100 previous phototherapy sessions in their lifetime.

### Interventions

- Oral vitamin D supplementation will be administered at 100,000 IU, mono-dose (cholecalciferol, UVEDOSE^®^), every 3 months (standard guidelines) for 2 years. UVEDOSE^®^ is marketed by NextPharma LIMAY. The placebo of oral vitamin D supplementation is developed and marketed by NextPharma LIMAY.
- Phototherapy: the NB-UVB regimen will include a period of escalation of 3 weeks (three sessions per week for 3 weeks) and after that every 2 weeks for 6 months in total (winter: October to March). For phototype II–III, NB-UVB is initiated at 0.2 J/cm^2^ per session and is increased in increments of 0.1 from session to session up to the ninth session. For maintenance (every 2 weeks), we will continue with the dosage of the ninth session (1.0 or 1.1 J/cm^2^). The dosage can be altered according to clinical tolerance. For phototype IV–V, NB-UVB is initiated at 0.3 J/cm^2^ per session and is increased in increments of 0.1 from session to session up to the ninth session. For maintenance (every 2 weeks), we will maintain the exposure level of the ninth session (1.1 or 1.2 J/cm^2^). The exposure can be altered according to clinical tolerance.

### Outcomes

#### Primary outcomes

The primary outcomes are (1) repeat measures of the Patient-Oriented Severity of Atopic Dermatitis Index (PO-SCORAD) severity score over 1 year and (2) cumulate consumption of TAT (number of tubes) during the winter; the outcomes will be tested following a hierarchical testing procedure. The choice of a patient-related score as the primary outcome will enable the capture of more data points without increasing the number of visits, and it is also coherent with the pragmatic design of the study.

#### Secondary outcomes

The secondary outcomes are repeat measures over 2 years for investigator-based severity scores (Eczema Area and Severity Index, Severity of Atopic Dermatitis Index, Investigator Global Assessment); patient-reported severity and quality of life scores (Patient-Oriented Eczema Measure, Dermatology Life Quality Index); serum vitamin D levels (25-(OH)-vitamin D); total IgE serum levels; weeks of satisfactory control; inter-visit cumulate consumption of TAT; and synthetic patient-reported satisfaction at the end of each winter.

Our trial includes all the recent core domains cited in the HOME recommendations [20].

### Recruitment

Particular attention will be given to recruiting a primary care population. Each region is organized from a university-based dermatology department into several ‘field study sites’ gathering several participating primary care dermatologists.

Meetings will be organized with primary care dermatologists to invite them to participate and to inform them on how the study will unfold. A trained study nurse will help them check their patients’ eligibility and plan the visits. The patients will have the possibility of receiving their phototherapy with their primary care dermatologist; scheduled visits will take place every 6 weeks.

### Sample size

We used the General Linear Multivariate Model Power & Sample Size (GLIMMPSE) software [30] to calculate the sample size for a repeat measure analysis with a desired power of at least 80%, a Type I error set at 0.05, and equal group sizes. The main predictor variable is phototherapy (yes or no) and the response variable is numerical (PO-SCORAD). Repeat measures are described as follows: 10 to 12 measures with time as the unit, and an equal distance between repeat numerical measures (every 4 weeks); we entered means for each time according to an anticipated evolution over time, and standard deviations of the response variable were set at 18. GLIMMPSE currently assumes that the standard deviation is constant across repeat measures. As we have some uncertainty about what standard deviation value to use, we used alternative values for variability and computed power for half the variance, the variance as specified, and twice the variance; this was also applied to means. GLIMMPSE automatically combines the sources of correlation into a final covariance matrix using a structured correlation based on the linear exponential auto-regressive model. The model describes a correlation that decreases monotonously with the distance between measures; the base correlation is set at 0.6 and the decay rate at 0.05. It can be noted that the minimum clinically significant difference for the PO-SCORAD score is indeed around 9, but we adopted a stance enabling the detection of a smaller difference, which means we are in an even better position to detect a clinically relevant difference.

We considered a main effect hypothesis test for the effect of a single predictor variable averaged across all other factors. We used the Hotelling–Lawley trace as the recommended statistical test for this kind of design, with a simple ‘between’ hypothesis and a complex ‘within’ hypothesis. We indeed plan to use a mixed model for our data analysis, and we will use the Wald test with Kenward–Roger degrees of freedom. It can be noted that the Hotelling–Lawley trace test for the general linear multivariate model coincides with the Wald test for the general linear mixed model.

On the basis of the sample size simulation (Additional file 1), we chose a total sample size of 340 subjects. This calculation was performed on the basis of a parallel-arm design; thus, using a crossover design, we considered we could halve the sample size. The assumption of no carry-over effect is allowed when planning a sample size for a crossover trial. For an intervention only in winter, we considered carryover would be minimized by design. Using a uniform and balanced crossover design, the treatment difference will not be aliased with sequence or period effects. However, an interaction of time and treatment should be taken into account, as we anticipate this could occur.

Power considerations in the area of repeat measures, crossover design and interaction between time and treatment are not straightforward. We therefore used GLIMMPSE, as for a two-arm trial. We realize that halving the sample size could appear simplistic and we therefore compared the sample size calculations between (1) a two parallel arm trial and (2) a crossover trial, considering only the last measure as the outcome, to check that the sample size reduction was not too optimistic. Finally, anticipating that 10–15% of patients will not complete the entire study (two 1-year sequences), the planned number of subjects to be enrolled is set at 200.

### Participant timeline

**Fig. 1:**
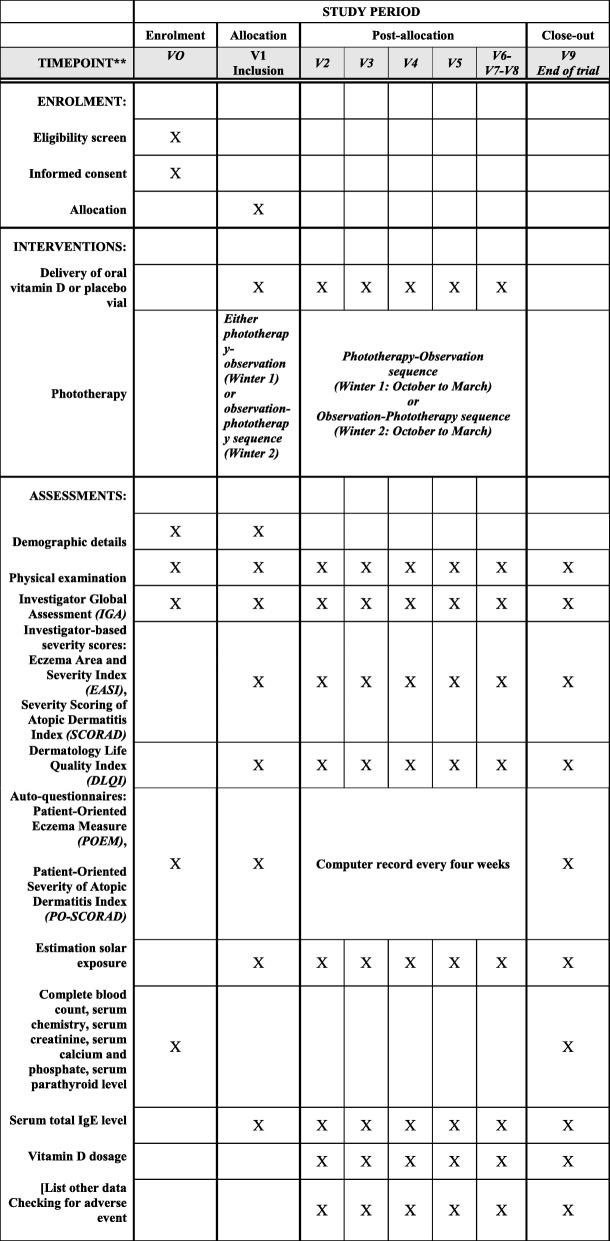
Overview of the study

Following the opening of screening in January, patients can enter the study at any time; they will go through a pre-screening period until they are randomized in September. We have chosen a common fixed period for inclusion to avoid confounding seasonality factors. During the pre-screening period, trial outcomes are collected but no treatment is given. Enrollment is preceded by a pre-screening period of 9 months to identify patients with inclusion criteria, and exclude, before randomization, any participants who are unlikely to be adherent. A patient will be in the trial for 2 years post-randomization (i.e., until September 2019 for those entering in September 2017). After the inclusion visit, visits will occur every 3 months over 2 years. Furthermore, each patient will complete the self-administered questionnaires including the PO-SCORAD at home through an application on their computer or phone, or otherwise on paper, every 4 weeks. The time schedule of enrolment and visits is provided in Table 1 and Fig. 1.

**Table 1:** Time schedule of enrolment and visits

Actions	Visit 0 Screening	Visit 1 Inclusion	Visits 2–8 Follow-up	Visit 9 End of trial
Written informed consent	X			
Web-based randomization		X		
Demographic details	X	X		
Medical history	X	X	X	X
Physical examination	X	X	X	X
Investigator Global Assessment	X	X	X	X
Eczema Area and Severity Index, Severity of Atopic Dermatitis Index		X	X	X
Quality of Life: Dermatology Life Quality Index		X	X	X
Patient-Oriented Severity of Atopic Dermatitis Index, Patient-Oriented Eczema Measure	X	X	Computer record every 4 weeks	X
Estimation of solar exposure		X	X	X
Complete blood count, serum chemistry, serum creatinine, serum calcium and phosphate, serum parathyroid level	X			X
Serum total IgE level		X	X	X
Vitamin D dosage		X	X	X
Delivery of oral vitamin D or placebo vial		X	X	
Checking for adverse events			X	X

### Assignment of interventions

#### Sequence generation and allocation concealment mechanism

Participants who meet the eligibility criteria and have signed informed consent will then be randomly assigned, in a 1:1 ratio, to either vitamin D or placebo, and within each arm to either the sequence UV–observation or the sequence observation–UV through an automated web-based system. A permuted block randomization scheme will be used, with stratification according to the clinical site, age (three age groups) and disease severity (two groups: moderate or severe). The list will be balanced by blocks of random size in order to ensure total unpredictability. Randomization can occur on the day of inclusion (Visit 1).

**Fig. 2:**
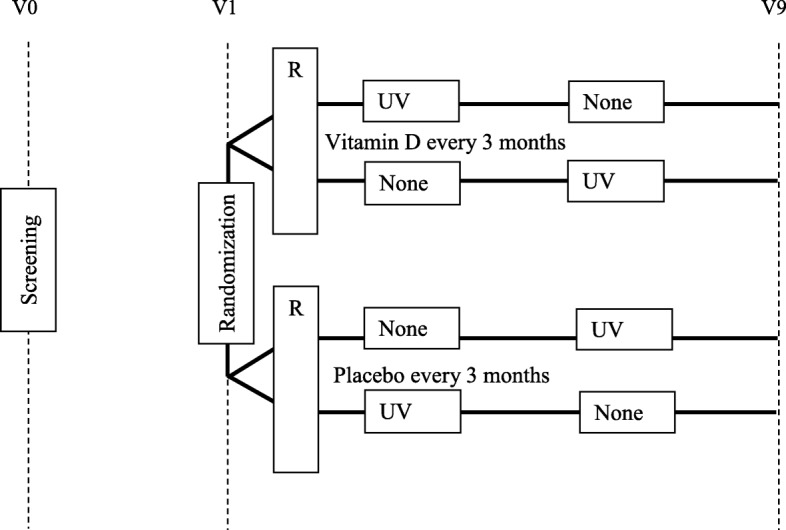
Figure SPIRIT

Since we offer phototherapy sessions exclusively during winter periods, the carry-over effect is not a concern in our study (Fig. 2).

#### Blinding

This study is conducted as open-label regarding phototherapy, since blinding cannot be implemented, but it is double-blind for vitamin D therapy.

Vitamin D: patients, investigators and outcome assessors will be blinded. Although it would have been possible to implement logistics with a blinded switch to placebo for patients reaching normal vitamin D levels, the absence of supplementation dose adaptation based on vitamin D serum level will help maintain the blinding.

Phototherapy: patients and investigators will not be blinded, and cannot be. We will address the problem of blinding with regard to phototherapy by masking the allocated treatment (phototherapy or observation) for the investigator in charge of scoring disease severity. However, some objective clues (e.g., tanning, if present) cannot be masked.

### Data collection, management, and analysis

#### Data collection methods

Table 1 shows data collection at inclusion and at follow-up visits.

#### Data management

All information required by the study protocol is to be recorded in the case report forms and an explanation is to be provided for any missing data. Data is to be collected progressively, as obtained, and recorded explicitly in the case report forms.

An electronic case report form (e-CRF) will be made available and data capture will be carried out in the centers using a web interface (Clinsight software, Ennov, Paris, France). Only an Internet connection and a browser are required. A document on how to use this tool will be provided for the investigators. The interface between the clinical research associate and the investigator will thus be provided, also making remote data collection and control possible. Data consistency tests will be incorporated in electronic format. An audit function is incorporated in the e-CRF, thus making it possible to follow any change in study data. This function also makes it possible to clearly identify the person who has made a change, with the date; comments can be added.

If requested, a hard copy will be printed at the end of the study, authenticated (dated and signed) by the investigator, and copies will be sent to the sponsor and archived.

The procedures applied are those used Rennes CHU and comply with Good Clinical Practice and the legal and regulatory requirements in force.

#### Statistical methods

The statistical issue for the primary outcomes is the analysis of repeat measures (up to 12 measures for each 1-year period, on the PO-SCORAD, a self-administered questionnaire the results of which are collected through an application on computer or phone, at home) applied to a crossover design (two 1-year periods, two sequences). When repeat measures are analyzed, there are many possible hypotheses that could be of interest. We focused on a main effect hypothesis test for the effect of a single predictor variable (treatment: phototherapy) averaged across all other factors, and the sample size calculation was designed accordingly. We are interested in the usual factors, namely ‘Treatment’ (phototherapy/no phototherapy) and ‘Time’. However, the nature of the design (crossover trial) needs to be taken into account. This means that we need to consider the following factors: ‘Period’ – first winter/second winter, ‘Order’ – phototherapy–no phototherapy or no phototherapy–phototherapy, and ‘Carry-over’. It was anticipated that vitamin D could act as a quantitative effect-modifier on phototherapy. We therefore decided to include an interaction term in the analysis. We will use a single model with the following main effects: Treatment-1 (phototherapy/no phototherapy), and Treatment-2 (vitamin D/placebo), Period, Order, and Carry-over terms as well as an interaction term between Treatment-2 and Treatment-1. As an interaction of Time and Treatment could occur, we plan to add an interaction term to the model. The other primary outcome, the cumulate consumption of TAT (number of tubes) during the winter, is a quantitative variable. It will be analyzed using a mixed model with the following main effects: Treatment-1 (phototherapy/no phototherapy), and Treatment-2 (vitamin D/placebo), Period, Order, and Carry-over terms as well as an interaction term between Treatment-2 and Treatment-1. As this outcome is a summary of winter consumption, no interaction of Time and Treatment will be added to this model. Adjustment will be made on the variables used in the allocation process (region – north vs. south), age as a continuous variable, and disease severity (moderate vs. severe).

For descriptive purposes, it is instructive to carry out an analysis based on a summary of the statistics (a quantity calculated from each curve), which can reflect important aspects of the problem at hand – we have chosen the area under the curve (AUC) and the mean amplitude of PO-SCORAD score ranges. Descriptive statistics (mean and standard deviation) of the PO-SCORAD for each visit will also be provided. Normal distribution is assumed in the modeling analysis for the primary outcomes (PO-SCORAD severity score over two 1-year periods; cumulate consumption of TAT during winter) and will be checked at this stage.

The main analysis will be based on the adjusted model and will follow the ‘intention to treat’ principle, namely all participants, as randomized, will be analyzed; outcome data obtained from all participants, regardless of protocol adherence, will be used. Mixed model techniques (PROC MIXED) will be applied.

The multiplicity adjustment strategy used is a hierarchical closed test procedure, where the primary endpoints are ordered as follows: (E1) repeat measures of the PO-SCORAD severity score over 1 year and (E2) cumulate consumption of TAT over winter. We will test E1 and E2 sequentially at the same two-sided level of 0.05; we will test E1 first, if it proves significant, will we test E2. A Wald test with Kenward–Roger degrees of freedom will be used.

No subgroup analyses nor interim analyses are planned.

For secondary analyses, we will use up to four measures per 1-year period, collected at each clinical visit (every 3 months), including investigator-based severity scores (Eczema Area and Severity Index, Severity of Atopic Dermatitis Index, Investigator Global Assessment), patient-reported severity and quality of life scores (Patient-Oriented Eczema Measure, Dermatology Life Quality Index), serum vitamin D levels, total IgE serum levels, weeks of satisfactory control, inter-visit cumulate consumption of TAT, and synthetic patient-reported satisfaction at the end of each winter.

All analyses will use procedures available in SAS software 9.4 (SAS Institute, Carry, NC, USA).

### Methods: Monitoring

#### Data monitoring

A clinical research technician will be responsible for coordinating the study; specifically, the logistics and monitoring of the study, the production of reports on progress, verifying that the e-CRFs are updated, dispatch of blood samples, and notification severe adverse events (AEs) to the sponsor. The technician will follow the Standardized Operating Procedures. The data monitoring technician will be dependent towards the sponsor and independent from the study investigators. Investigators will make the data available to the persons in charge of monitoring, quality checks, or audit of the study documents, and they will provide individual data that is strictly necessary for such checking, in compliance with the legislative and regulatory conditions in force (articles L.1121–3 and R.5121–13 in the French public health code).

#### Adverse events

Any serious AEs, whether or not related to the medical object under study, expected or unexpected, must be reported within 24 h by the investigator to the sponsor on a “Serious AE” form, on which should be specified the date of occurrence, the severity rating, the intensity, the relationship with the treatment evaluated (or the study), and the outcome. All other AEs will be reported on the “AE” form in the case report form specifying the date of occurrence, the description, the severity, the duration, the method of resolution, the causal relationship, and the decisions made. The sponsor will report all suspected unexpected serious adverse drug reactions to Eudravigilance (the European pharmacovigilance database), the French Health Products Agency (Agence de Sécurité du Médicament et des Produits de Santé), and the investigators, within the regulatory time periods for reporting.

#### Auditing

The investigators agree to undergo quality assurance audits conducted by the sponsor as well as inspections performed by the competent authorities. All data and all documents and reports can be subjected to audits and regulatory inspections without limitation in relation to medical confidentiality.

### Ethics and dissemination

#### Ethics approval for the research

The sponsor and the investigators undertake to conduct this study in compliance with French law no. 2004–806 of August 9, 2004, and with Good Clinical Practice and the Helsinki Declaration (Ethical Principles for Medical Research involving Human Subjects, Tokyo, 2004). The study will be conducted in accordance with this protocol. Excluding emergency situations requiring specific therapeutic actions, the investigators will observe the protocol in all respects, particularly regarding consent and the notification and follow-up of serious AEs.

The protocol was approved on 18/08/15 by the “Comité de protection des Personnes” and received authorization from the French Health Products Agency on 27/07/15.

#### Consent

Patients will be completely and openly informed, in terms that are understandable to them, of the objectives and constraints of the study, of the possible risks incurred, of the required measures of supervision and safety, of their right to refuse to participate in the study, and of the possibility of withdrawing their consent at any time.

All this information is contained in an information and consent form given to the patient. The patients’ free, informed and written consent will be collected by the investigator, or a doctor representing them prior to final inclusion in the study. A copy of the information and consent form signed by both parties will be given to the patient; the investigator will keep the original.

#### Confidentiality and access to data

In compliance with the conditions of confidentiality of data to which individuals in charge of quality control of biomedical studies have access (article L.1121–3 in the French public health code), and with conditions pertaining to confidentiality of information, in particular concerning the type of investigational medical design, the tests, subjects in the study, and the results obtained (article R. 5121–13 in the French public health code), individuals having direct access will take all necessary precautions to ensure confidentiality of the information.

#### Dissemination policy

The data provided by the investigating centers will be analyzed by INSERM CIC 1414 Rennes. A written report on the results will be submitted to the sponsor and published on ClinicalTrials.gov.

Scientific presentations and reports corresponding to the study will be drafted under the responsibility of the coordinating investigator of the study with the agreement of the investigators in charge. Rules for publication will follow international recommendations [31]. In accordance with law no. 2002–303 of March 4, 2002, patients will be informed, at their request, of the overall results of the study.

### SPIRIT guidelines

This protocol has been written in accordance with the Standard Protocol Items: Recommendations for Interventional Trials (SPIRIT) guidelines. The SPIRIT checklist is provided in Additional file 2.

## Discussion

AD is a highly prevalent disease in both adults and children, with acknowledged unmet needs, in particular in the LTC of the disease, which has not been widely evaluated in published studies. Novel and safe therapeutic strategies are needed for long-term AD control and a potentially effective low-cost therapeutic strategy for LTC is essential for patients and public health.

The international, multi-professional initiative HOME aims to standardize and validate AD outcome measurements. LTC has been defined as a core domain to be assessed in AD studies, but the corresponding outcome measure has not yet been established and future research is needed [20]. The HOME initiative encourages the conduct of trials on LTC of AD, and cites two broad approaches to explore LTC; the first is the assessment of weeks during which the disease is well-controlled and the second is to perform serial measurements of the other three core domains (signs, symptoms, and quality of life). HOME has validated a duration of more than 3 months for future AD trials assessing LTC. Because TAT cannot be entirely withdrawn, testing ‘add-on’ therapies for LTC of AD needs both the collection of AD severity measures and an evaluation of TAT consumption. In this trial, our proposal is to evaluate AD severity by serial measurements of investigator- and patient-reported severity scores over a 2-year period, alongside TAT consumption.

Since both phototherapy and vitamin D supplementation are good candidates for LTC of AD disease activity, our proposal is to study these two treatments jointly. We will therefore use a factorial design to assess the effects of phototherapy and vitamin D supplementation on LTC within the same study; since solar exposure (and to a lesser extent artificial UV exposure under phototherapy) favors vitamin D synthesis, this design has the potential to explore the interaction between phototherapy and vitamin D supplementation in disease control and serum vitamin D levels in winter and all-year round. We have chosen a crossover design for phototherapy to adjust on personal characteristics (phototherapy and sensitivity to UV exposure being major determinants of phototherapy efficacy) [32–34]. We chose a fixed and common period for inclusion to avoid confounding by seasonality. While patients will be able to sign their written consent at any time in the pre-screening period, they will be randomized in September and we cannot exclude that some patients may drop out of the study before the randomization. The alternative would be to randomize patients throughout the year and to take into account seasonal effects, but the approach to measure this would have been complex in a study with pragmatic options and long-termfollow-up. Since we provide phototherapy sessions exclusively during winter periods, a carry-over effect is not a concern in our study.

Our trial provides the possibility of reducing topical anti­inflammatory treatment use. Further evaluation will be needed if the exploratory trial on phototherapy opens up new avenues (novel phototherapy, pro-active strategy).

As we are conducting a long-term study, we plan the following procedures to minimize attrition bias: firstly, any participant who is recommended systemic immunosuppressants will discontinue the study; secondly, our trial includes strong pragmatic options [29, 34–36] such as primary care population, standard vitamin D supplementation that is not adapted to serum levels, ‘as usual’ TAT use, and no recommendations for changes in lifestyle; thirdly, the trial includes logistic support to ensure adherence such as trained study nurses in the field facilitating participant adherence; and, finally, it will test new maintenance therapies superimposed on usual care. It can be noted that ‘add-on’ therapy trials on asthma have better rates of adherence [37].

## Trial status

The protocol is currently recruiting in 16 French centers. The first patient was included on September 30, 2015, and recruitment is anticipated to end in September 2020.

## Additional files

Additional file 1: Details on sample size calculation. (DOCX 75 kb)

Additional file 2: SPIRIT 2013 checklist: recommended items to address in a clinical trial protocol and related documents. (DOC 98 kb)

## Abbreviations

AD: atopic dermatitis
AE: adverse event
e-CRF: electronic case report form
GLIMMPSE: general linear multivariate model power & sample size
HOME: Harmonising Outcome Measures for Eczema
LTC: long-term control
PO-SCORAD: Patient-Oriented Severity of Atopic Dermatitis Index
SAS: statistical analysis system
SCORAD: Severity Scoring of Atopic Dermatitis Index
TAT: topical anti-inflammatory treatments
UV: ultraviolet
