# Supplementary material for: A new phototherapy regimen during winter as an add-on therapy, coupled with oral vitamin D supplementation, for the long-term control of atopic dermatitis: study protocol for a multicentre, randomized, crossover, pragmatic trial – the PRADA trial
Source: Trials. 2019 Mar 25;20:184. doi: 10.1186/s13063-019-3276-9 (PMC6434814; doi:10.1186/s13063-019-3276-9)
Supplement: Supplementary file 1 — Details on sample size calculation. (DOCX 75 kb) [file 13063_2019_3276_MOESM1_ESM.docx]

**Additional file 1:** Details on sample size calculation

The options selected in GLIMMPSE were as follows:

1. solving for sample size

2. desired power: 0.80, 0.85, 0.90

3. model

a. clustering: participant, no additional clustering

b. predictor: treatment (phototherapy)

c. covariate: none

d. response variable: score (PO-SCORAD)

e. repeated measure: dimension: time; type: numeric; number: 10; spacing: equal

f. group sizes: equal

4. hypothesis

a. hypothesis type: main effect; factor of interest: between-participant factor: treatment

b. statistical test: Hotelling-Lawley test

c. type 1 error rate: 0.05

5. means

a. means

40 39 38 37 36 35 34 33 32 32

40 40 40 40 40 40 40 40 40 40

b. scale factor for means: 0.9, 1.0, 1.1

6. variability

a. within-participant variability

i. variability across time: base correlation: 0.6; decay rate: 0.05 (LEAR model)

ii. variability across response (score) : standard deviation: 18 (unstructured correlation)

b. scale factors for variability: 0.5, 1, 2

Matrix used in calculation:


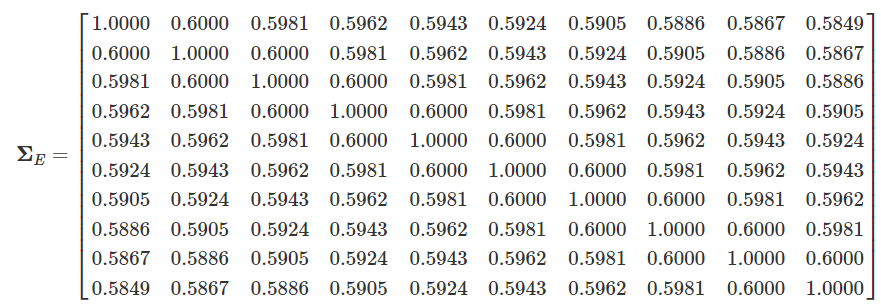


Results for GLIMMPSE sample size simulation for a main effect hypothesis following the aforementioned assumptions:

| Actual Power | Total Sample Size | Mean Scale | SD Scale |
| --- | --- | --- | --- |
| 0.800 | 336 | 1.0 | 1.0 |
| 0.850 | 384 | 1.0 | 1.0 |
| 0.901 | 450 | 1.0 | 1.0 |

Script up-loaded into GLIMMPSE:

{"uuid":[],"name":null,"gaussianCovariate":false,"solutionTypeEnum":"SAMPLE_SIZE","participantLabel":null,"viewTypeEnum":"GUIDED_MODE","confidenceIntervalDescriptions":null,"powerCurveDescriptions":null,"alphaList":[{"idx":0,"alphaValue":0.05}],"betaScaleList":[{"idx":0,"value":0.9},{"idx":1,"value":1.1},{"idx":2,"value":1}],"sigmaScaleList":[{"idx":0,"value":0.5},{"idx":1,"value":1},{"idx":2,"value":2}],"relativeGroupSizeList":[{"idx":0,"value":1},{"idx":0,"value":1}],"sampleSizeList":[],"statisticalTestList":[{"idx":"0","type":"HLT"}],"powerMethodList":[],"quantileList":[],"nominalPowerList":[{"idx":0,"value":0.8},{"idx":1,"value":0.85},{"idx":2,"value":0.9}],"responseList":[{"idx":0,"name":"Score"}],"betweenParticipantFactorList":[{"idx":0,"predictorName":"Treatment","categoryList":[{"idx":0,"category":"Active"},{"idx":0,"category":"Placebo"}]}],"repeatedMeasuresTree":[{"idx":0,"node":0,"parent":0,"repeatedMeasuresDimensionType":"NUMERICAL","numberOfMeasurements":10,"spacingList":[{"idx":1,"value":1},{"idx":2,"value":2},{"idx":3,"value":3},{"idx":4,"value":4},{"idx":5,"value":5},{"idx":6,"value":6},{"idx":7,"value":7},{"idx":8,"value":8},{"idx":9,"value":9},{"idx":10,"value":10}],"dimension":"Time"}],"clusteringTree":[],"hypothesis":[{"idx":1,"type":"MAIN_EFFECT","betweenParticipantFactorMapList":[{"type":"NONE","betweenParticipantFactor":{"idx":0,"predictorName":"Treatment","categoryList":[{"idx":0,"category":"Active"},{"idx":0,"category":"Placebo"}]}}],"repeatedMeasuresMapTree":[]}],"covariance":[{"idx":0,"type":"LEAR_CORRELATION","name":"Time","standardDeviationList":[{"idx":0,"value":1},{"idx":0,"value":1},{"idx":0,"value":1},{"idx":0,"value":1},{"idx":0,"value":1},{"idx":0,"value":1},{"idx":0,"value":1},{"idx":0,"value":1},{"idx":0,"value":1},{"idx":0,"value":1}],"rho":0.6,"delta":0.05,"rows":10,"columns":10,"blob":{"data":[[1,0.6,0.5980874585828169,0.5961810135234212,0.5942806453892516,0.5923863348096896,0.5904980624758616,0.5886158091404424,0.5867395556174594,0.584869282782096],[0.6,1,0.6,0.5980874585828169,0.5961810135234212,0.5942806453892516,0.5923863348096896,0.5904980624758616,0.5886158091404424,0.5867395556174594],[0.5980874585828169,0.6,1,0.6,0.5980874585828169,0.5961810135234212,0.5942806453892516,0.5923863348096896,0.5904980624758616,0.5886158091404424],[0.5961810135234212,0.5980874585828169,0.6,1,0.6,0.5980874585828169,0.5961810135234212,0.5942806453892516,0.5923863348096896,0.5904980624758616],[0.5942806453892516,0.5961810135234212,0.5980874585828169,0.6,1,0.6,0.5980874585828169,0.5961810135234212,0.5942806453892516,0.5923863348096896],[0.5923863348096896,0.5942806453892516,0.5961810135234212,0.5980874585828169,0.6,1,0.6,0.5980874585828169,0.5961810135234212,0.5942806453892516],[0.5904980624758616,0.5923863348096896,0.5942806453892516,0.5961810135234212,0.5980874585828169,0.6,1,0.6,0.5980874585828169,0.5961810135234212],[0.5886158091404424,0.5904980624758616,0.5923863348096896,0.5942806453892516,0.5961810135234212,0.5980874585828169,0.6,1,0.6,0.5980874585828169],[0.5867395556174594,0.5886158091404424,0.5904980624758616,0.5923863348096896,0.5942806453892516,0.5961810135234212,0.5980874585828169,0.6,1,0.6],[0.584869282782096,0.5867395556174594,0.5886158091404424,0.5904980624758616,0.5923863348096896,0.5942806453892516,0.5961810135234212,0.5980874585828169,0.6,1]]}},{"idx":0,"type":"UNSTRUCTURED_CORRELATION","name":"__RESPONSE_COVARIANCE__","standardDeviationList":[{"idx":0,"value":18}],"rho":-2,"delta":-1,"rows":1,"columns":1,"blob":{"data":[[1]]}}],"matrixSet":[{"idx":0,"name":"beta","rows":2,"columns":10,"data":{"data":[[40,39,38,37,36,35,34,33,32,32],[40,40,40,40,40,40,40,40,40,40]]}}]}
